# Production of Anti-Cancer Agent Using Microbial Biotransformation

**DOI:** 10.3390/molecules191016684

**Published:** 2014-10-16

**Authors:** Changhyun Roh, ChanKyu Kang

**Affiliations:** 1 Division of Biotechnology, Advanced Radiation Technology Institute (ARTI), Korea Atomic Energy Research Institute (KAERI), 1266, Sinjeong-dong, Jeongeup, Jeonbuk 580-185, Korea; 2 Ministry of Environment, Daegu Regional Environmental Office, Government Complex, Hwaam-ro, Dalseo-Gu, Daegu 704-841, Korea

**Keywords:** biotransformation, screening, production, fermentation

## Abstract

Microbial biotransformation is a great model system to produce drugs and biologically active compounds. In this study, we elucidated the fermentation and production of an anti-cancer agent from a microbial process for regiospecific hydroxylation of resveratrol. Among the strains examined, a potent strain showed high regiospecific hydroxylation activity to produce piceatannol. In a 5 L (w/v 3 L) jar fermentation, this wild type *Streptomyces* sp. in the batch system produced 205 mg of piceatannol (*i.e.*, 60% yields) from 342 mg of resveratrol in 20 h. Using the product, an *in vitro* anti-cancer study was performed against a human cancer cell line (HeLa). It showed that the biotransformed piceatannol possessed a significant anticancer activity. This result demonstrates that a biotransformation screening method might be of therapeutic interest with respect to the identification of anti-cancer drugs.

## 1. Introduction

Resveratrol (3,4,5-transhydroxystilbene), one of the most widely studied polyphenols produced by plants, is of growing scientific interest, since it enhances the health-related qualities in humans [1,2,3]. It is well-known for its presence in red wine. As a component of grape skin (up to 0.1%), extracts containing resveratrol have been combined with the colored anthocyanin into red wine during fermentation. The health effect of red wine is believed to result from its high concentration of polyphenols, including resveratrol [3,4,5]. A number of researchers have reported the multiple beneficial effects of resveratrol in humans, including anti-carcinogenic and low-density lipoproteins [3]. Piceatannol (3,3,4,5-trans-trihydroxystilbene), an analogue of resveratrol, also displays a wide spectrum of biological activities; however, relatively less research attention has been given to this compound [6]. Piceatannol has been found in various plants, including grapes, passion fruit, white tea, and Japanese knotweed. Besides antioxidative effects, piceatannol exhibits potential anticancer properties as suggested by its ability to suppress proliferation of a wide variety of tumor cells, including leukemia, lymphoma, and cancers of the breast, prostate, colon, melanoma and apoptosis in colorectal cancer [7,8]. Piceatannol and resveratrol are hard soluble in water but dissolve in ethanol and dimethyl sulfoxide. The stilbene-based structure of these compounds consists of two phenolic rings linked by a styrene double bond to generate 3,4',5-trihydroxystilbene and 3,5,3',4'-tetrahydroxystilbene, respectively. Although the presence of the double-bond facilitates trans- and cis-isomeric forms ((*E*)- and (*Z*)-diastereomers, respectively), the trans-isomers are sterically the more stable forms [9]. Regiospecific hydroxylation of aromatic compounds by chemical synthesis is difficult and involves diverse reaction steps. Regiospecific microbial hydroxylation at a non-activated carbon atom of aromatic compounds is attractive and remarkable biosynthesis. The introduction of hydroxyl groups into target compounds by the use of microorganisms represents an attractive alternative to conventional chemical synthesis [10,11]. In this study, *Streptomyces* sp. Strain SB-14 screened using an enrichment culture showed high activity for regiospecific hydroxylation of resveratrol. The selective modification of resveratrol by the microbial process is a powerful challenge to the production of the anti-cancer compound, piceatannol. To the best of our knowledge, this is first report to show the production of piceatannol using microbial biotransformation.

## 2. Results and Discussion

### 2.1. Screening and Identification of the Microorganism for Regiospecific Hydroxylation of Resveratrol

**Figure 1:**
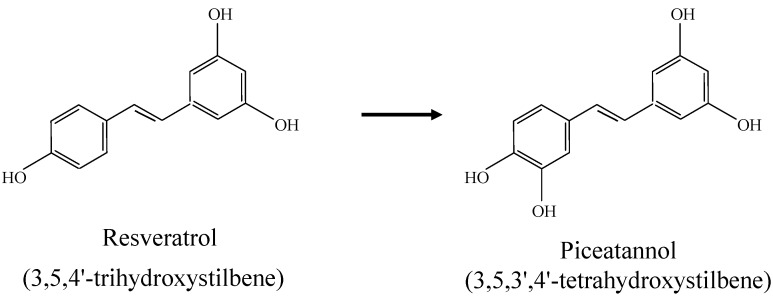
Molecular structures of trans-resveratrol and piceatannol. The conversion of *trans*-resveratrol to piceatannol is catalyzed by *Streptomyces* sp. Strain SB-14.

After several rounds of enrichment culture, a strain with regiospecific resveratrol hydroxylation activity was found. The whole-cell reaction using the screened microorganism yielded mass peaks and chromatogram with the same retention time. The molecular structure of piceatannol represents the regiospecific hydroxylation of resveratrol shown in Figure 1. The genomic DNA of strain SB-14 screened was extracted using a commercial genomic DNA extraction kit (Genomictree); PCR-mediated amplification of the 16S rRNA gene and sequencing of the purified PCR product were carried out as previously described [12]. The nearly complete sequence of the 16S rRNA gene (1230 nt) was compiled using SeqMan software (DNASTAR). The 16S rRNA gene sequences of the related taxonomy were obtained from GenBank and were edited using the program BioEdit [13]. Multiple alignments were performed with the program CLUSTAL X [14]. Evolutionary distances were calculated with the Kimura two-parameter model [15]. Phylogenetic trees were constructed using neighbor-joining [16] and maximum-parsimony [17] methods in MEGA3 [18].

**Figure 2:**
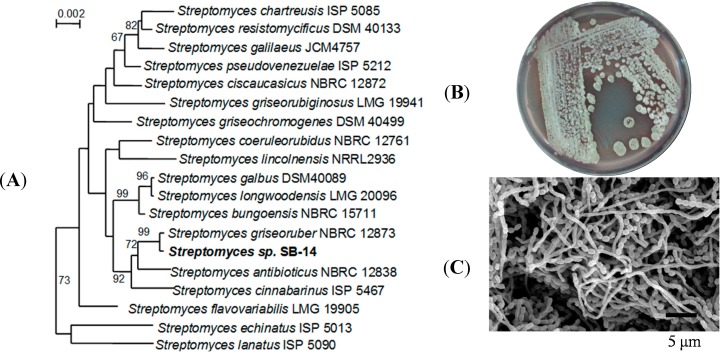
(**A**) Neighbor-joining phylogenetic tree based on 16S rRNA gene sequences showing the relationships between strain SB-14 and the type strains of recognized Streptomyces species. Numbers at branch points are bootstrap values (percentages of 1000 replications); only values > 50% are shown. Bar, 0.002 substitutions per nucleotide position. The (**B**) *Streptomyces* sp. Strain SB-14 screened (**C**) Scanning electron microscopy (SEM) image.

In the neighbor-joining tree based on 16S rRNA gene sequences, strain SB-14 belonged to the glade formed by members of the genus Streptomyces in the family Actinomycetes (Figure 2A). In the phylogenetic relationships on the basis of the 16S rDNA sequence, the strain SB-14 was closely related to *Streptomyces griseoruber* NBRC 12873 (98.94%). The sequences are sense 5'-GTTTTAGAGTTTTGGACT-3', antisense 5'-CGTGACGTGACGGGCGGT-3'. The predominant quinones were MK-9(H8) and MK-9(H6). The major cellular fatty acids were anteiso-C15:0 (39.18%), anteiso-C17:0 (20.32%) and C16:0 (11.71%). The G+C content of SB-14 was 74.3%. The color of the substrate mycelium of SB-14 was red and aereial mycelium was gray on the ISP2 medium (Figure 2B). The image of mycelium was analyzed by scanning electron microscopy (Figure 2C).

**Figure 3:**
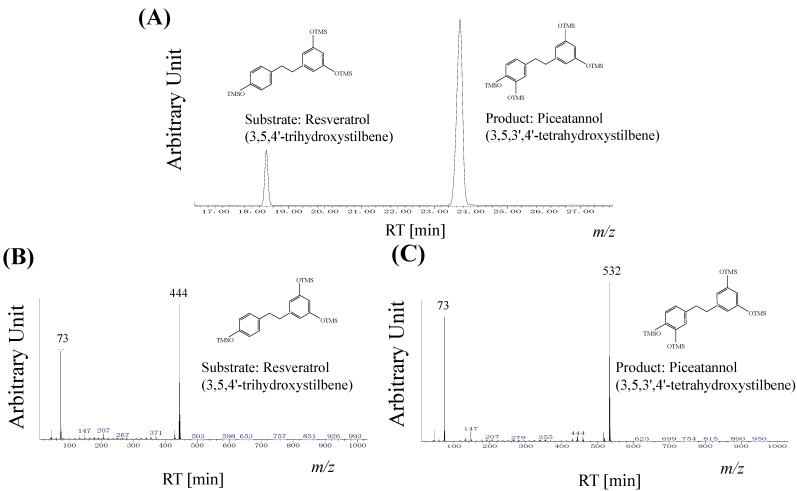
Gas Chromatography-Mass Spectrometry (GC-MS) analysis of resveratrol and piceatannol produced by *Streptomyces* sp. Strain SB-14. (**A**) GC chromatograms of resveratrol and piceatannol, Retention times: resveratrol, 18.4 min; piceatannol, 23.7 min (**B**) Mass spectra of resveratrol (**C**) Mass spectra of piceatannol.

In the regiospecific hydroxylated product, piceatannol detected in the whole cell reaction was identified. The analysis of the product from resveratrol was confirmed by GC chromatograms (Figure 3A). For further mass spectrum, the substrate and product were analyzed using MS, which are in good agreement with the hydroxylated form, and were observed at *m/z* 444 for 3,5,4'-trihydroxystilbene, 532 for 3,5,3',4'-tetrahydroxystilbene (Figure 3B,C).

### 2.2. Biotransformation for Production of Piceatannol

**Figure 4:**
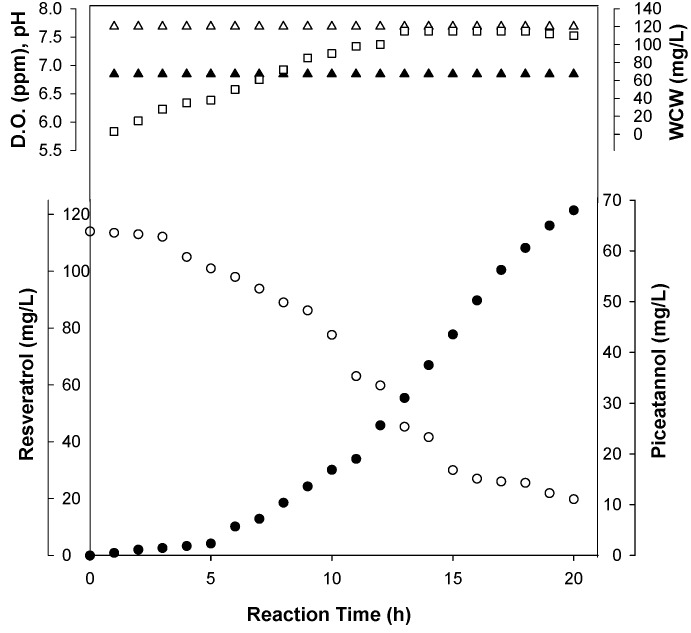
Biotransformation for the production of piceatannol using *Streptomyces* sp. Strain SB-14.

Time-dependent reaction profiles of the formation of piceatannol from resveratrol were obtained using whole cell biotransformation. When 0.5 mM of resveratrol was used, *ca.* 90% of the substrate was consumed within 24 h. In a 5 L (w/v 3 L) jar fermentation, the *Streptomyces* sp. screened produced 205 mg of piceatannol (*i.e.*, 60% yields) from 342 mg of resveratrol in 20 h (Figure 4).

### 2.3. Anti-Cancer Activity Using Biotransformed Piceatannol

The viability of the cells was checked by an MTT assay, which detects the percentage of irreversibly damaged cells after treatment with the indicated inhibitor, piceatannol. By adding piceatannol at a concentration of 30 μM the percentage of viable cells decreased to 45%.

**Figure 5:**
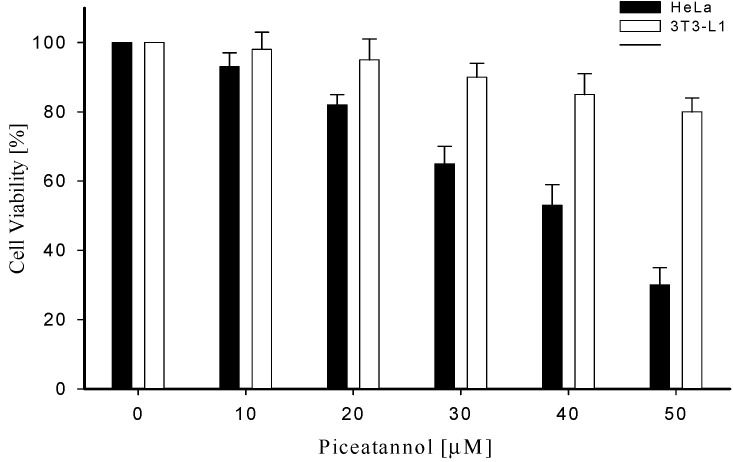
Anti-cancer activity using biotransformed piceatannol. 3T3-L1 was used as a normal animal cell line.

A significant reduction of viable cells with piceatannol was detected at a high inhibitor concentration of 50 μM. Interestingly, a biotransformed piceatannol did not inhibit normal animal cell, 3T3-L1 (Figure 5). In this study, we screened the *Streptomyces* sp. Strain SB-14 to have regiospecific hydroxylation activity to produce piceatannol from resveratrol. From GC-MS analysis, we confirmed that the reaction product is a regiospecific hydroxylated form of the substrate. The GC-MS result for the product from the substrate is consistent with the expected molecular weight of the hydroxylated form with the same retention time on a chromatograph. Using 100 g/L of *Streptomyces* sp. Strain SB-14 wet cell mass, piceatannol was produced to a level of about 68 mg/L in a bio-catalytically active whole cell reaction.

## 3. Experimental Section

### 3.1. Chemicals

Resveratrol, piceatannol and all chemicals were purchased from Sigma-Aldrich Chemical Co. (St. Louis, MO, USA). And, *N*,*O*-bis(trimethylsily)trifluoroacetamide was purchased from Sigma-Aldrich Chemical Co, St. Louis, MO, USA. All other chemicals were of the highest grade available.

### 3.2. Screening of Strain and Culture Conditions

An enrichment culture was performed to isolate a novel microorganism which had the ability to catalyze the hydroxylation of resveratrol. After field soil samples from a soybean farm (5 g) were mixed with 50 mL phosphate-buffered saline (PBS), the mixture was filtered using filter paper (alpha cotton cellulose, 110-mm diameter; Advantec, Fukuoka, Japan). The filtered sample (100 μL) was inoculated into a 10 mL minimal M9-resveratrol medium with 0.2% (w/v) resveratrol as a carbon source instead of glucose. Cultures were grown at 30 °C under aerobic conditions. After several rounds of enrichment cultures, the culture media were diluted, spread on an M9-resveratrol minimal medium or ISP2 agar plate containing 1% (w/v) malt extract, 0.4 (w/v) yeast extract, 0.4% (w/v) glucose, and incubated at 30 °C for 20 h. More than one hundred colonies were selected randomly, based on differences in morphology and color. The selected strains were subsequently cultured in 3 mL of M9-resveratrol medium at 30 °C. Cultured cells were stored at −80 °C for further study. After the cells were harvested and washed using PBS, whole-cell reactions were performed to find strains with resveratrol hydroxylation activity. Strains with high resveratrol hydroxylation activity were selected. 16S rRNA sequencing (Genomictree Co., Daejeon, Korea) was performed to identify the screened microorganism. A subculture of the strains was generated at 28 °C, 220 g for 3 days in a test tube containing 5 mL of ISP2 medium. From 1 mL of the subculture, cultivation was carried out in a 250 mL conical flask containing 50 mL of the medium. For a preparative fermentation, it was performed in a 5:l (w/v 3:l) jar fermentation.

### 3.3. Microbial Biotransformation

The cells harvested from a culture broth of *Streptomyces* sp. Strain SB-14 were washed twice with a potassium phosphate buffer (50 mM, pH 7.2). After centrifugation (13,000 *g*, 10 min), 100 mg of cells (wet wt.) was added in 900 μL of a potassium phosphate buffer (100 mM, pH 7.2) with 100 μL of resveratrol (0.5 mM in DMSO). The total reaction volume was 1 mL and shaken for 24 h at 28 °C. After 12 h, the reactant was extracted with ethylacetate (JUNSEI, Kyoto, Japan). The extracted sample was evaporated in a centrifugal vacuum concentrator (BioTron, Puchon, Korea) and dissolved in methanol (MERCK, Darmstadt, Germany).

### 3.4. Gas Chromatography (GC)/Mass Spectrometry (MS) Analysis

For GC/MS analysis, the reactions were converted to their TMS (trimethylsilyl) derivatives by incubating for 20 min at 60 °C with BSTFA (*N*,*O*-bis(trimethylsily)trifluoroacetamide). GC/MS was carried out on a Finnigan MAT system (Gas chromatograph model GCQ, HP 19091J-433) connected to an ion trap mass detector. The TMS-derivatives were analyzed using a nonpolar capillary column (5% phenyl methyl siloxane capillary 30 m × 250 μm i.d., 0.25 μm film thickness, HP-5) with a linear temperature gradient (60 °C 5 min, 50 °C·min^−1^ to 250 °C, held for 10 min, 1 °C·min^−1^ to 300 °C, and held for 3 min). The injector port temperature was 250 °C. The scan spectrum was 100–600 *m/z* and the mass spectrum was obtained by electron impact ionization at 70 eV. The derivatized samples of resveratrol and piceatannol were separated using the selected ion mode (SIM).

### 3.5. Isolation of Product, Piceatannol

For purified piceatannol, the fermented sample was applied to Prep-HPLC under the following conditions: column, Alltech Econosil C18 (22 by 250 mm, 5 μm particle size, Alltech Associate, Inc., Deerfield, IL, USA); UV detection, 320 nm; flow rate, 5.0 mL·min^−1^; mobile phase, acetonitrile/water (v/v) for 60 min followed by a 10%–40% acetonitrile linear gradient, 60% acetonitrile for 40 min and followed by 70% acetonitrile for 30 min.

### 3.6. Anti-Cancer Activity Assay

HeLa cells were cultured in an RPMI 1640 medium containing 10% (v/v) fetal calf serum, 2 mM glutamine (Life Technologies, Inc., Carlsbad, CA, USA), penicillin (100 U/mL) and streptomycin (100 μg/mL) in a humidified 5% CO_2_ atmosphere. For inhibitor treatment, 5–7 × 10^6^ HeLa cells were incubated with piceatannol in 0.5% dimethyl sulfoxide (DMSO) for 24 h at indicated concentrations. Control cells were treated with 0.5% DMSO. Cell viability was assessed by an MTT (3-(4,5-dimethylthiazol-2-yl)-2,5-diphenyltetrazolium bromide) assay (Sigma-Aldrich, St. Louis, MO, USA), which is based on the ability of a mitochondrial dehydrogenase from viable cells to oxidize the tetrazolium rings of the pale yellow MTT and form dark blue formazan crystals, which are largely impermeable to cell membranes, and, therefore, accumulate within healthy cells. The number of surviving cells is directly proportional to the concentration of the formazan produced. Cells were incubated with the indicated inhibitors at different concentrations (0 or 50 μM) for 24 h. Then a solution of MTT in phosphate-buffered saline (PBS) was added to each well to a final concentration of 0.5 μg/μL. After 4 h incubation, dark blue formazan was solubilized with 100 μL DMSO. Absorbance was measured at 590 nm using UV-vis spectroscopy.

## 4. Conclusions

The biocatalytic reactions can be performed by screened whole cell system, which may be microorganisms. It is a significant fact that the screening of a wide variety of microorganism living in environments is an efficient approach to pave the way for novel drugs and bioactive compounds. Thus, microbial processes have become a good model system to develop an industrial biotransformation system to produce drugs and biologically active compounds. Kim group showed a hydroxylation of resveratrol by inhibition of tyrosinase from *Streptomyces avermitilis* MA4680 [19]. Moreover, the Kino group demonstrated a regioselective synthesis of piceatannol from resveratrol using recombinant flavin monooxygenase enzyme [20]. Interestingly, this is the first report elucidating the fermentation and production of an anti-cancer compound using a microbial process screened for regiospecific hydroxylation of resveratrol. This study has a significant scope in that the biotransformation result provides one initiative example such that a regiospecific hydroxylated compound from resveratrol showing more potent biological activity can be produced at a large scale using microbial biotransformation.
